# Assessing agreement among non-invasive indicators for inspiratory effort during pressure support ventilation

**DOI:** 10.3389/fmed.2025.1561017

**Published:** 2025-03-05

**Authors:** Wen-Yi Lv, Shuai Liu, Linlin Zhang, Jian-Xin Zhou

**Affiliations:** ^1^Department of Critical Care Medicine, Beijing Tiantan Hospital, Capital Medical University, Beijing, China; ^2^Emergency and Critical Care Center, Clinical and Research Center on Acute Lung Injury, Beijing Shijitan Hospital, Capital Medical University, Beijing, China

**Keywords:** mechanical ventilation, pressure support ventilation, inspiratory effort, PMI, ΔPocc, P0.1

## Abstract

**Background:**

During pressure support ventilation (PSV), the accuracy of non-invasive indicators in diagnosing high or low inspiratory effort has been validated. However, the correlation and agreement of these indicators remain unclear. This study aims to investigate the correlation and agreement among non-invasive inspiratory effort indicators, and to compare characteristics of inspiratory effort in neurocritical and non-neurocritical patients.

**Methods:**

This was a single-centre prospective observational study. We collected three non-invasive inspiratory effort indicators, pressure muscular index (PMI), the maximal negative swing of airway pressure during expiratory occlusion (ΔPocc), and the airway occlusion pressure during the first 100ms (P0.1). Cutoff values for these indicators derived from esophageal pressure-time product (PTPmus) were chosen for this study. The correlation and agreement of these indicators were analyzed using Spearman’s rank correlation test and linear weighted Kappa analysis. Characteristics of PSV settings and inspiratory effort in neurocritical and non-neurocritical patients were compared.

**Results:**

Ninety-seven patients were enrolled in this study. Correlation analysis showed a moderate correlation between PMI and ΔPocc (rho = −0.524, *p* < 0.001), ΔPocc and P0.1 (rho = 0.588, *p* < 0.001), while no correlation between PMI and P0.1 (rho = −0.140, *p* = 0.172). There was a moderate agreement between ΔPocc and P0.1 (*k* = 0.459, *p* < 0.001), a fair agreement between PMI and ΔPocc (*k* = 0.362, *p* < 0.001), but no agreement between PMI and P0.1 (*k* = 0.134, *p* = 0.072). The correlation of these indicators was similar in neurocritical patients compared with non-neurocritical patients, but agreement was poor.

**Conclusion:**

The study showed that PMI and ΔPocc had moderate correlation and fair agreement, ΔPocc and P0.1 had moderate correlation and agreement, while PMI and P0.1 had no correlation and agreement.

## 1 Introduction

Pressure support ventilation (PSV) is one of the most used ventilation modes for mechanically ventilated patients, particularly during the weaning process (1). In clinical practice, lung and diaphragm protective ventilation strategy is recommended to prevent high and low inspiratory efforts (2, 3), which can frequently occur during PSV (4, 5), and lead to complications affecting patient outcomes and increasing hospitalization expenses (2, 6–8).

The esophageal pressure-time product (PTPmus), calculated from esophageal pressure, is considered the gold standard for inspiratory effort (2, 9). However, this parameter is usually used for research purposes and not for routine clinical use because of the need for invasive procedures, special equipment, and complexity of calculations.

Recently, several non-invasive inspiratory effort indicators, such as pressure muscular index (PMI), the maximal negative swing of airway pressure during expiratory occlusion (ΔPocc), and the airway occlusion pressure during the first 100 ms (P0.1), have been found to have good ability to detect high or low inspiratory effort and can be directly measured on the ventilator screen without additional tools (10–14). In previous studies, high and low inspiratory effort cutoff values for PMI, ΔPocc, and P0.1 were derived from PTPmus (5, 14, 15). Although previous studies have validated the diagnostic accuracy of these indicators (5, 10, 13–16), the agreement among these measurements still remains underexplored.

Neurocritical care patients admitted to the intensive care unit (ICU) often require artificial airway and/or ventilation support due to impaired consciousness, poor airway protective ability, and damaged respiratory drive and conduction pathways. These patients face a higher risk of difficult weaning, delayed extubation, extubation failure, and tracheostomy (17–19). The characteristics of inspiratory effort in neurocritical patients and whether they differ from those in non-neurocritical patients are unknown.

The aim of this study was to investigate the correlation and agreement of non-invasive inspiratory effort indicators (PMI, ΔPocc and P0.1) in patients in the intensive care unit (ICU). The secondary aim was to compare characteristics of inspiratory effort in neurocritical and non-neurocritical patients.

## 2 Materials and methods

### 2.1 Study participants

This was a single-centre prospective observational study that enrolled adult patients aged 18–85 years. A total of 97 patients admitted to the ICU of Beijing Tiantan Hospital using PSV between January and August 2024 were included in this study. Patients younger than 18 years old, older than 85 years old, unable to measure the non-invasive inspiratory effort due to the ventilator incapability, receiving extracorporeal life support, or palliative care were excluded from the study. Neurocritical care patients specifically refer to the adult patients admitted to ICU for primary and/or secondary brain injuries. This study was presented according to STROBE guidelines (Supplementary File) and approved by the Institutional Review Board of Beijing Tiantan Hospital with approval number: KY2023-206-02 (January 2, 2024), and the informed consent was obtained in each participant.

### 2.2 Study design

All participants underwent three non-invasive inspiratory effort measurements, PMI, ΔPocc, and P0.1. To reduce the interference from continuous measurements and improve accuracy, each type of measurement was conducted three times, with an interval of at least 1 min between each measurement. The average of the three results was used for data analysis. A total of nine measurements were conducted in one patient.

The definitions and cutoff values for high and low inspiratory effort remain inconsistent across different studies (3, 20–25). PMI, ΔPocc, and P0.1 cutoff values derived from PTPmus were chosen for our study due to PTPmus is considered the gold standard for inspiratory effort (2, 9). According to Yang YL’s study, when high and low effort was defined as PTPmus > 200 and <50 cmH_2_O⋅s⋅min^1^, the cutoff values of PMI were 2.1 and 0 cmH_2_O, ΔPocc were −8.4 and −5.7 cmH_2_O, P0.1 were −2.2 and −1.1 cmH_2_O, respectively (5).

***PMI.*** During PSV, after an end-inspiratory airway occlusion, the airway pressure (Paw) will reach a plateau (26). With the ventilator’s screen freeze function activated, the PMI can be measured on the ventilator screen as the difference between the plateau Paw and the peak Paw (11).

***ΔPocc.*** During an expiratory airway occlusion, the patient’s inspiratory effort against the occluded airway causes the Paw to deviate, reaching a minimum value (Pnadir), and then returning to the baseline (13). The difference between Pnadir and the positive end-expiratory pressure (PEEP) on the ventilator screen can be calculated as ΔPocc.

***P0.1.*** P0.1 is directly measured by the ventilators. Previous studies have showed that the P0.1 displayed by various ventilators could accurately reflects the P0.1 calculated using a reference method (14).

The ventilators involved in this study were Dräger Series (Dräger, Lubeck, Germany), Maquet Servo-i (Maquet Critical Care, Solna, Sweden), the Mindray SV Series (Myriad BioMedical Electronics, Shenzhen, China) and Prunus Padus 8 (PB Medical, Shenzhen, China).

### 2.3 Statistical analysis

All data were analyzed using the Statistical Program for Social Sciences (SPSS) version 25 (IBM Corporation, Armonk, NY, USA). Categorical variables were presented as counts and percentages, while continuous variables were expressed as means with standard deviations (SD) or medians with interquartile ranges (IQR). The Shapiro–Wilk test was used to assess the normality of the non-invasive inspiratory effort data, which was found to be non-normal distributed. The correlation and agreement were tested using Spearman’s rank correlation test and linear weighted Kappa analysis (k), respectively. The Spearman’s rank correlation coefficient (rho) classified the strength of the correlation as follows: negligible (rho = 0.00–0.09), weak (rho = 0.10–0.39), moderate (rho = 0.40–0.69), strong (rho = 0.70–0.89), and very strong (rho = 0.90–1.00) (27). The strength of agreement based on the weight Kappa coefficient (k) was interpreted as: slight (*k* = 0–0.20), fair (*k* = 0.21–0.40), moderate (*k* = 0.41–0.60), substantial (*k* = 0.61–0.80), and excellent (*k* = 0.81–1.00) (28). Neurocritical and non-neurocritical data were compared using independent samples *t*-tests or Mann-Whitney U nonparametric tests. For categorical variables, tests were performed using the chi-square test or Fisher’s exact test. All tests were two-sided, and a *p* < 0.05 was considered statistically significant.

## 3 Results

A total of 97 patients (mean age, 63 ± 17.4 years; 63.9% male) were recruited in the study. The baseline demographics were shown in Table 1. According to the PTPmus-based PMI, ΔPocc, and P0.1 cutoff values, data were categorized into high, normal, and low inspiratory effort groups, respectively. The results showed that 34.0%, 52.6%, and 48.5% of patients may have low inspiratory effort, while 10.3%, 28.9%, and 20.6% of patients may have high inspiratory effort. 51.5% of the observation points showed the same classification between PMI and ΔPocc measurements, 39.2% between PMI and P0.1, and 58.8% between ΔPocc and P0.1.

**TABLE 1:** Patients’ characteristics.

Patient characteristics	All (*n* = 97)	Neurocritical (*n* = 47)	Non-neurocritical (*n* = 50)	*p*-value*
Male	62 (63.9%)	30 (63.8%)	30 (64.0%)	0.986
Age (years)	63 (17.4)	55 (15.0)	70 (16.8)	**<0.001**
Patient weight (kg)	70 (13.1)	72 (12.4)	68 (13.7)	0.742
Patient height (cm)	168 (6.9)	167 (6.7)	168 (7.2)	0.117
GCS at ICU admission	9 (4.5)	8 (4.2)	11 (4.5)	**0.006**
APACHE II at ICU admission	17 (7.9)	15 (7.4)	20 (7.6)	**<0.001**
SOFA on the day before study	5 (3.4)	5 (2.6)	6 (3.9)	0.156
Surgery	67 (69.1%)	41 (87.2%)	26 (52.0%)	**<0.001**
Analgesia	46 (47.4%)	27 (57.4%)	19 (38.0%)	0.055
Sedation	35 (36.1%)	28 (59.6%)	17 (34.0%)	**0.012**
Systolic blood pressure (mmHg)	135 (18)	133 (20)	136 (17)	0.427
Diastolic blood pressure (mmHg)	70 (14)	74 (14)	66 (14)	**0.004**
Heart rate (beats/min)	89 (16)	91 (17)	88 (14)	0.842
Respiratory rate (breaths/min)	19 (6)	19 (6)	19 (5)	0.423
Temperature (°)	36.9 (0.6)	37.1 (0.7)	36.7 (0.6)	**0.004**
Spo_2_	99 (2.1)	99 (1.8)	99 (2.3)	0.319
VT (mL)	495 (153)	484 (134)	500 (169)	0.564
MV (L)	8.6 (2.7)	8.1 (2.25)	9.0 (3.1)	0.080
VT/PBW (mL/kg)	8.1 (2.5)	7.9 (2.3)	8.2 (2.7)	0.721
**The latest chest imaging**				
Pneumonia	55 (56.7%)	27 (57.4%)	28 (56.0%)	0.886
Heavy lung markings	39 (40.2%)	16 (34.0%)	23 (46.0%)	0.230
Pleural effusion	34 (35.1%)	15 (31.9%)	19 (38.0%)	0.530
**The latest arterial blood gas**				
pH	7.4 (0.07)	7.4 (0.08)	7.4 (0.06)	0.683
Pao_2_ (mmHg)	123.6 (46.5)	122.6 (51.3)	124.5 (42.0)	0.440
Paco_2_ (mmHg)	36.7 (7.0)	37.5 (7.7)	36.1 (6.2)	0.322
HCO_3–_ (mmol/L)	25.1 (4.3)	25.4 (3.5)	24.8 (4.9)	0.499
Lac (mmol/L)	1.7 (0.85)	1.7 (0.69)	1.6 (0.98)	0.221
Type of artificial airway				**<0.001**
Oral intubation	61 (62.9%)	33 (70.2%)	28 (56.0%)	
Tracheostomy	7 (7.2%)	7 (14.9%)	–	
Nasal intubation	29 (29.9%)	7 (14.9%)	22 (44.0%)	
**PSV settings**				
Pressure support (cmH_2_O), median (IQR)	9 (8–10)	8 (7–9)	10 (8–12)	**<0.001**
PEEP (cmH_2_O), median (IQR)	5 (5–6)	5 (5–6)	5 (5–6)	0.131
FiO_2_ (%), median (IQR)	40 (35–40)	40 (35–40)	40 (35–40)	0.446
**Inspiratory effort**				
PMI (cmH_2_O)	0.5 (2.32)	0.5 (1.00)	0.5 (3.10)	0.140
ΔPocc (cmH_2_O)	6.5 (4.75)	6.2 (3.82)	6.8 (5.51)	0.960
P0.1 (cmH_2_O)	1.4 (1.14)	1.2 (0.95)	1.6 (1.27)	0.096

*Neurocritical and non-neurocritical patients’ data were compared. APACHE, Acute Physiology and Chronic Health Evaluation; FiO_2_, Fraction of Inspired Oxygen; GCS, Glasgow Coma Scale; HCO_3–_, Bicarbonate Ion; ICU, Intensive Care Unit; IQR, Interquartile Range; Lac, Lactic Acid; MV, Minute Ventilation Volume; PaCO_2_, Arterial Partial Pressure of Carbon Dioxide; PaO_2_, Arterial Partial Pressure of Oxygen; PEEP, Positive End-Expiratory Pressure; PBW, Predicted Body Weight; PMI, Pressure Muscular Index; P0.1, Airway Occlusion Pressure during the First 100ms; PSV, Pressure Support Ventilation; SOFA, Sequential Organ Failure Assessment; SpO_2_, Oxygen Saturation Measured by Pulse Oximetry; VT, Tidal Volume; ΔPocc, The Maximal Negative Swing of Airway Pressure during Expiratory Occlusion.

Compared to non-neurocritical patients, neurocritical patients were younger (55 vs 70 years, *p* < 0.001), had more postoperative patients (87.2% vs 52%, *p* < 0.001), lower GSC scores (8 vs 11, *p* = 0.006), lower APACHE II scores (15 vs 20, *p* < 0.001), more sedation use (59.6% vs 34%, p = 0.012), more tracheotomized patients (14.9% vs 0, *p* < 0.001), and lower pressure support (8 vs 10 cmH2O, *p* < 0.001) (Table 1). In neurocritical patients, based on PMI, ΔPocc, and P0.1 cutoff values, the proportions diagnosed with low inspiratory effort were 23.4, 51.1, and 57.4%, respectively, and the proportions with high inspiratory effort were 2.1, 25.5, and 17.0%, respectively.

The correlation and agreement analysis were shown in Table 2. In all patients, a moderate correlation was found between PMI and ΔPocc (rho = −0.524, *p* < 0.001), ΔPocc and P0.1 (rho = 0.588, *p* < 0.001), while no correlation between PMI and P0.1 (rho = −0.140, *p* = 0.172). There was a moderate agreement between ΔPocc and P0.1 (*k* = 0.459, *p* < 0.001), a fair agreement between PMI and ΔPocc (*k* = 0.362, *p* < 0.001), but no agreement between PMI and P0.1 (*k* = 0.134, *p* = 0.072).

**TABLE 2:** Correlation and Agreement of PMI, ΔPocc, and P0.1.

Variables	rho^1^	*p*-value	k^2^	*p*-value
**All patients (n = 97)**				
PMI-Δpocc	−0.524	**<0.001**	0.362	**<0.001**
PMI-P0.1	−0.140	0.172	0.134	0.072
Δpocc-P0.1	0.588	**<0.001**	0.459	**<0.001**
**Neurocritical (n = 47)**				
PMI-Δpocc	−0.504	**<0.001**	0.173	**0.041**
PMI-P0.1	−0.249	0.091	0.030	0.733
Δpocc-P0.1	0.667	**<0.001**	0.415	**<0.001**
**Non-neurocritical (n = 50)**				
PMI-Δpocc	−0.509	**<0.001**	0.505	**<0.001**
PMI-P0.1	−0.100	0.489	0.245	**0.025**
Δpocc-P0.1	0.538	**<0.001**	0.497	**<0.001**

^1^The correlation were tested using Spearman’s rank correlation test (rho).

^2^The agreement were tested using linear weighted Kappa analysis (k). PMI, Pressure Muscular Index; P0.1, Airway Occlusion Pressure during the First 100ms; ΔPocc, The Maximal Negative Swing of Airway Pressure during Expiratory Occlusion; Pmus, inspiratory muscle pressure; PTPmus/min, Pmus-time product per minute.

Correlations among PMI, ΔPocc, and P0.1 were similar between neurocritical and non-neurocritical patients (Table 2). The agreement of inspiratory effort diagnosis was inferior in neurocritical patients (k_*PMI–*Δ*Pocc*_ = 0.173, *p* = 0.041; k_*PMI–P0.1*_ = 0.030, *p* = 0.733, k_Δ*Pocc–P0.1*_ = 0.415, *p* < 0.001) than in non-neurocritical patients (k_*PMI–*Δ*Pocc*_ = 0.505, *p* < 0.001; k_*PMI–P0.1*_ = 0.245, *p* = 0.025, k_Δ*Pocc–P0.1*_ = 0.497, *p* < 0.001) (Table 2).

## 4 Discussion

In this study, the results indicated that PMI and ΔPocc had moderate correlation and fair agreement, ΔPocc and P0.1 had moderate correlation and moderate agreement, while PMI and P0.1 had no correlation and agreement. The correlation of non-invasive inspiratory effort indicators was similar in neurocritical patients compared with non-neurocritical patients, but agreement was poor. To our knowledge, this is the first study describing characteristics of inspiratory effort in neurocritical patients.

In our study, a larger proportion of patients had inspiratory effort outside the recommended physiologic range, which is similar to previous findings. According to the PTPmus-based PMI, ΔPocc, and P0.1 cutoff values, the results showed that 34.0%, −52.6% patients may have low inspiratory effort, while 10.3%–28.9% patients may have high inspiratory effort. Previous studies have also shown that over-assistance under PSV is not uncommon (4, 29).

Previous studies have shown that PMI, ΔPocc, and P0.1 have good accuracy in detecting high and low inspiratory effort in mechanical ventilation patients. However, the definitions and cutoff values for high and low inspiratory effort remain inconsistent across different studies (3, 20–25). For detecting high inspiratory effort [defined as changes in transdiaphragmatic pressure [ΔPdi] > 12 cmH_2_O (10), changes in esophageal pressure [ΔPes] > 12 cmH_2_O (16), Pmus > 10 cmH_2_O (5, 13), or PTPmus > 200 cmH_2_O⋅s⋅min^1^ (5, 14, 15)], PMI cutoff values range between 2.1 and 3.8 cmH_2_O (5, 16) (AUC 0.93, sensitivity 68%–88%, specificity 81%–92%); ΔPocc cutoff values between 8.4 and 17.9 cmH_2_O (5, 10, 13, 16) (AUC 0.86–0.93, sensitivity 80%–100%, specificity 67%–84%); and P0.1 cutoff values between 2.0 and 4.0 cmH_2_O (5, 10, 15, 16, 30) (AUC 0.73–0.95, sensitivity 52%–100%, specificity 72%–94%). On the other hand, for detecting low inspiratory effort [defined as ΔPdi < 3 cmH_2_O (10), ΔPes < 5 cmH_2_O (16), Pmus < 5 cmH_2_O (5), or PTPmus < 50 cmH_2_O⋅s⋅min^1^ (5, 14)], PMI cutoff values range between −0.4 and 0.0 cmH_2_O (5, 16) (AUC 0.89–0.95, sensitivity 30%–96%, specificity 86%–95%), ΔPocc cutoff values between 5.7 and 7.5 cmH_2_O (5, 10, 16) (AUC 0.93–0.97, sensitivity 60%–88%, specificity 85%–95%), and P0.1 cutoff values between 0.9 and 1.3 cmH_2_O (5, 10, 16, 30) (AUC 0.87–0.93, sensitivity 65%–100%, specificity 61%–89%).

Due to the lack of uniform definitions for high and low inspiratory effort, various criteria have been used across studies, resulting in different cutoff values. In our study, only the gold standard PTPmus-based cutoff values of PMI, ΔPocc, and P0.1 were selected for the diagnosis of high or low inspiratory effort. However, only one study derived cutoff values for PMI, ΔPocc, and P0.1 based on the PTPmus (5). These suggest that the cutoff values used in our study may not accurately diagnose high or low inspiratory effort, which may lead to discrepancies in diagnosis based on PMI, ΔPocc, and P0.1, which explains their limited agreements.

In our study, we found that the correlations among PMI, ΔPocc, and P0.1 were similar in neurocritical patients compared with non-neurocritical patients, but agreement was poor. This may be because cutoff values provided by previous studies were not specific to neurocritical care patients, the thresholds used in our study may not be applicable to these population. In our study, nearly half of the study participants were neurocritical care patients, who differ from non-neurological patients in their need for mechanical ventilation, not only due to pulmonary or cardiac causes, but also brain-related factors such as impaired consciousness, decreased airway protection, and disrupted respiratory drive (17, 18, 31–33).

Respiration is a complex process involving multiple organs and systems. Respiratory movements in mammals are driven by rhythmic neural activity generated spatially and functionally by a brainstem neural network consisting of the respiratory central pattern generator (RCPG) (34). Neurological influences on respiration also include chemoreceptor modulation (35), airway protective reflexes (32), respiratory functional plasticity (36), and neurotransmitter remodeling of respiratory patterns (37). These complex respiratory regulatory systems are susceptible to brain damage. Neurocritical care patients may exhibit different inspiratory effort cutoff values compared to non-neurological patients. The cutoff value of the noninvasive inspiratory effort indicators in neurocritical care patients may require further study.

In our study, we averaged three separate measurements of PMI, ΔPocc, and P0.1, with at least a 1-min interval between each measurement, to reduce the interference from continuous measurements and improve accuracy. The patient’s inspiratory effort may vary from breath to breath. The inspiratory effort values obtained from PMI, ΔPocc, and P0.1 did not represent the same breath, which may weaken correlation and agreement.

There are several limitations to our study. First, the study included a small number of patients and measurement points. Since the study only included patients from a single medical center and nearly half of the study participants were neurocritical care patients, the findings might not be generalizable to other patients. Second, our study did not include the gold standard for comparison. We did not perform oesophageal manometry, so the accuracy of the diagnosis based on PMI, POCC, and P0.1 is not known. Finally, the order of measurement of noninvasive inspiratory effort indicators was fixed, which increased the bias of our study.

In conclusion, this study compared PMI, ΔPocc, and P0.1 as non-invasive tools for measuring inspiratory effort in ICU patients. The study showed that PMI and ΔPocc had moderate correlation and fair agreement, ΔPocc and P0.1 had moderate correlation and agreement, while PMI and P0.1 had no correlation and agreement.

## Data Availability

The raw data supporting the conclusions of this article will be made available by the authors, without undue reservation.
