# Supplementary material for: Assessing agreement among non-invasive indicators for inspiratory effort during pressure support ventilation
Source: Front Med (Lausanne). 2025 Mar 5;12:1561017. doi: 10.3389/fmed.2025.1561017 (PMC11919886; doi:10.3389/fmed.2025.1561017)
Supplement: Supplementary file 1 [file Table_1.pdf]

STROBE Statement—Checklist of items that should be included in reports of ***cross-sectional studies***

	Item No	Recommendation	Page No: Line No
**Title and abstract**	1	(*a*) Indicate the study's design with a commonly used term in the title or the abstract	01: 4-12
		(*b*) Provide in the abstract an informative and balanced summary of what was done and what was found	01: 21-51
Introduction			
Background/rationale	2	Explain the scientific background and rationale for the investigation being reported	02: 119-154
Objectives	3	State specific objectives, including any prespecified hypotheses	02: 155-159
Methods			
Study design	4	Present key elements of study design early in the paper	02: 186-221
Setting	5	Describe the setting, locations, and relevant dates, including periods of recruitment, exposure, follow-up, and data collection	02: 167-181
Participants	6	(*a*) Give the eligibility criteria, and the sources and methods of selection of participants	02: 167-176
Variables	7	Clearly define all outcomes, exposures, predictors, potential confounders, and effect modifiers. Give diagnostic criteria, if applicable	02: 193-216
Data sources/ measurement	8*	For each variable of interest, give sources of data and details of methods of assessment (measurement). Describe comparability of assessment methods if there is more than one group	02: 226-236 04:379-391
Bias	9	Describe any efforts to address potential sources of bias	NA
Study size	10	Explain how the study size was arrived at	02: 166-170
Quantitative variables	11	Explain how quantitative variables were handled in the analyses. If applicable, describe which groupings were chosen and why	02:193-201
Statistical methods	12	(*a*) Describe all statistical methods, including those used to control for confounding	02: 226-236 04:379-391
		(*b*) Describe any methods used to examine subgroups and interactions	04: 386-389
		(*c*) Explain how missing data were addressed	NA
		(*d*) If applicable, describe analytical methods taking account of sampling strategy	NA
		(*e*) Describe any sensitivity analyses	NA
Results			
Participants	13*	(a) Report numbers of individuals at each stage of study—eg numbers potentially eligible, examined for eligibility, confirmed eligible, included in the study, completing follow-up, and analysed	04: 396
		(b) Give reasons for non-participation at each stage	NA

		(c) Consider use of a flow diagram	NA
Descriptive data	14*	(a) Give characteristics of study participants (eg demographic, clinical, social) and information on exposures and potential confounders	04:396-398; 408-414
		(b) Indicate number of participants with missing data for each variable of interest	NA
Outcome data	15*	Report numbers of outcome events or summary measures	04: 399-435
Main results	16	(*a*) Give unadjusted estimates and, if applicable, confounder-adjusted estimates and their precision (eg, 95% confidence interval). Make clear which confounders were adjusted for and why they were included	NA
		(*b*) Report category boundaries when continuous variables were categorized	04: 399-407
		(*c*) If relevant, consider translating estimates of relative risk into absolute risk for a meaningful time period	NA
Other analyses	17	Report other analyses done—eg analyses of subgroups and interactions, and sensitivity analyses	04: 428-435
Discussion			
Key results	18	Summarise key results with reference to study objectives	04:440-447
Limitations	19	Discuss limitations of the study, taking into account sources of potential bias or imprecision. Discuss both direction and magnitude of any potential bias	05: 527-538
Interpretation	20	Give a cautious overall interpretation of results considering objectives, limitations, multiplicity of analyses, results from similar studies, and other relevant evidence	04-05: 448-526
Generalisability	21	Discuss the generalisability (external validity) of the study results	05: 530-533
Other information			
Funding	22	Give the source of funding and the role of the funders for the present study and, if applicable, for the original study on which the present article is based	05: 576-582

*Give information separately for exposed and unexposed groups.

Note: An Explanation and Elaboration article discusses each checklist item and gives methodological background and published examples of transparent reporting. The STROBE checklist is best used in conjunction with this article (freely available on the Web sites of PLoS Medicine at http://www.plosmedicine.org/, Annals of Internal Medicine at http://www.annals.org/, and Epidemiology at http://www.epidem.com/). Information on the STROBE Initiative is available at www.strobe-statement.org.
